# *Actinidia macrosperma* C. F. Liang (a Wild Kiwi): Preliminary Study of Its Antioxidant and Cytotoxic Activities

**DOI:** 10.1155/2012/180262

**Published:** 2011-10-24

**Authors:** Yin Lu, Xiangtao Du, Lidan Lai, Hao Jin

**Affiliations:** College of Biological and Environmental Engineering, Zhejiang Shuren University, Hangzhou 310015, China

## Abstract

The antioxidant potential of *Actinidia macrosperma* C. F. Liang (Actinidiaceae) was investigated in vitro for total phenolic content, along with total antioxidant activity (TAA), 1,1-diphenyl 2-picryl hydrazyl (DPPH), and lipid peroxidation (LP). The results indicated that different polarity extracts of *A. macrosperma* exhibit different biological activities, which depends mainly on the presence of phenolic compounds. The antioxidant activity was in the following decreasing order: MeOH extract > EtOAc extract > aqueous extract > CHCl_3_ extract > Hexane extract. Moreover, the cytotoxic activity of this plant by MTT dye assay using SMMC-7721 has been determined also. The hexane, EtOAc, and CHCl_3_ extracts showed cytotoxicity in a dose-dependent manner. Methanol and aqueous extracts, however, showed weak activities in this test. And a very significant cytotoxic activity, not significantly different from the positive control of quercetin, was observed in CHCl_3_ extract.

## 1. Introduction 

Free radicals and other reactive oxygen species (ROS) are mostly considered to be associated with pathogenesis and be responsible for the initiation or/and development of many diseases such as atherosclerosis, inflammation, cancer, hypertension, ischemia-reperfusion, autoimmune diseases, aging and age-related diseases [1–4]. Antioxidants may have an important role in preventing or alleviating chronic diseases by reducing the oxidative damage to cellular components caused by free radicals and ROS [5]. Although synthetic antioxidants were often used to protect against free radicals by scavenging reactive oxygen or ending radical chain reactions [6, 7], some such as *tert*-butyl hydroxyanisole (BHA) and *tert*-butyl hydroxytoluene (BHT) have been shown to have toxic and carcinogenic effects on human health [8–10]. Powerful endogenous antioxidant defences are thought to be augmented by dietary antioxidants, and so much of the protective effect of fruits and vegetables has been attributed to their high content of antioxidants [11]. A number of reports on the isolation and testing of plant-derived antioxidants in the maintenance and improvement of health and wellness have been described during the past decade. Hence, investigations of antioxidants are now focused on natural antioxidative compounds, which constitute a broad range of substances including phenolic or nitrogen-containing compounds and carotenoids [12–14]. In spite of large structure diversity, these compounds all share the same chemical pattern—one or more phenolic groups, for which they react as hydrogen donors in absorbing and neutralizing free radicals, quenching singlet and triplet oxygens, or decomposing peroxides [15]. Therefore, the substitution of synthetic antioxidants by natural ones and the screening of plant species for identifying new antioxidants in the maintenance and improvement of health and wellness become critically important in recent years.

**Figure 1:**
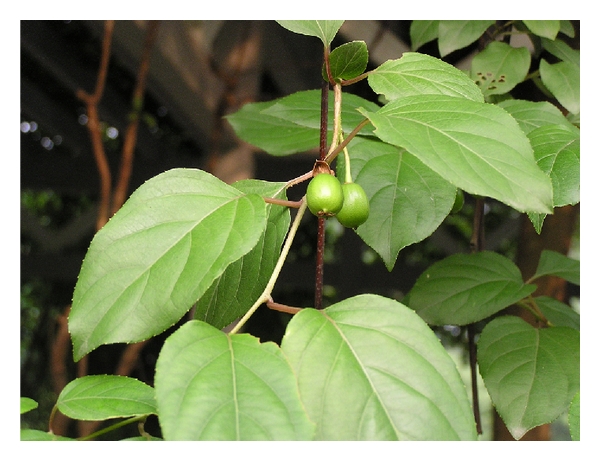
Picture of wild plants of *A. macrosperma. *

*Actinidia macrosperma* (shown as Figure 1), a naturally wild kiwi and endemic to eastern China, is popularly called “Cat Ginseng” because of the function attracting cats to use the plant as a stimulant and a healer for wounds [16]. It is a midsized deciduous, scandent shrub producing white flowers in spring (April-May) and orange fruits in late September and grows wildly in hillsides below 800 m above sea level, at piedmont, and moist margins of forest or along brook [17]. The roots and stems of the plant have been extensively employed to treat various ailments like leprosy, abscess, rheumatism, arthritis inflammation, jaundice, and abnormal leucorrhea [18] and were also useful for the treatment of cancers, especially those of lung, liver, and digestive system [16, 19]. 

Several clinical trials on patients receiving chemotherapy or radiotherapy have found that *A. macrosperma* significantly improved appetite, alleviated weakness, increased weight, and stabilized white blood cell counts, NK cells, IL-2, and CD4/CD8 ratio [20]. Others have shown it could improve the quality of life of the subjects by enhancing physical function and healthy transition (respondents' amount of change in their health in general over a 1-year period) without any adverse side effects in the patients with cancers of gastric, esophageal, liver, and lung [18]. The injection isolated from this plant had in vitro antitumor efficacy to three liver cancer cell lines (H_22_, CBRH-7919, and SMMC-7721) [21]. Its active fraction also showed the activity against the transplanted H22 in mouse by use of influencing cell cycle (arrest at G0-G1 phase and decrease at S phase) and inducing apoptosis [22]. However, information regarding its active components and biological activities remains unclear so far.

In recent years, we have conducted a series of research projects focusing on *A. macrosperma *[23–27], which was traditionally used in China folk medicine and commercially sold as natural remedy. The objectives of this paper were to investigate its antioxidant potential in vitro and to determine its cytotoxic activity in human hepatoma (SMMC-7721) cells. Because the current botanical products used unextracted material, different extraction methods were used as a means of improving activity. It would be also of interest to find whether there was any correlation between phenolic contents and activities (antitumor and antioxidant). It should complement to previously known therapeutic value and improve the popularization of this somehow overlooked common herb species.

## 2. Subjects and Methods

In the present study, four different assays, total phenolic content, DPPH, FRAP, and TAB were used in vitro to evaluate the antioxidant activity of *A. macrosperma* extracts comparing to that of the known antioxidants, based on the reaction of specific reagent of each method with electron donating or hydrogen radical (H^∙^) producing antioxidant compounds. Moreover, cytotoxicity by MTT-dye assay using SMMC-7721 (human hepatoma cell lines) with IC_50_ (50% inhibitory concentration) were determined also.

### 2.1. Plant Material and Extraction

Fresh *A. macrosperma *were collected from the hills of the Fuyang region of Zhejiang Province, China, in April 2010. The sample was identified by the Lab of Plant Systematic Evolution and Biodiversity, Zhejiang University, and a voucher specimen (SP. No: A2010006) has been deposited at the Herbarium of Zhejiang University (HZU).

The stems of *A. macrosperma* were air-dried and made into coarse powder. The powder (200 g) was extracted successively with hexane, ethyl acetate (EtOAc), and methanol (MeOH) at 60°C in a Soxhlet apparatus until the refluxed solvent became colorless. The extracts were separately evaporated to dryness at 40~50°C in a rotary vacuum evaporator. The yields were 2.9%, 3.6%, and 2.4% (w/w), respectively. A hot water extract was prepared by boiling 100 g of the powdered material in 1000 mL distilled water for 15 min with continuous stirring and filtering through muslin to remove all solids. The filtrate was then evaporated under reduced pressure and lyophilized. The yield of the aqueous extract was 24.7% with reference to dry starting material. For preparation of the chloroform (CHCl_3_) extract, the sample (200 g) was soaked in chloroform (2000 mL) at room temperature for 7 days. The extract was evaporated to dryness as described above to give a 1.9% yield. All extracts were stored at −20°C until use.

### 2.2. Determination of Antioxidative Activity

#### 2.2.1. Amount of Total Phenolic Content

The amount of phenolic compounds in the extracts of *A. macrosperma* was determined using Folin-Ciocalteus reagent according to the method of Singleton et al. 200 *μ*L of each extract (1.0 mg/mL) dissolved in methanol was mixed with 1.0 mL of Folin-Ciocalteu reagent (previously diluted 1 : 10 v/v with water). Five minutes later, 800 *μ*L of Na_2_CO_3_ (7.5%) solution was added to the mixture. The mixture was shaken for 2 h at room temperature, and absorbance was measured at 765 nm. The concentrations of total phenolic compounds in each extract were expressed as gallic acid equivalents in milligrams per gram extract by reference to the gallic acid standard calibration curve. 

#### 2.2.2. DPPH Radical Assay

Free radical scavenging activitiy of the extracts on the stable radical DPPH (1,1-diphenyl-2-picryl-hydrazil) was estimated by the method of Brand-Williams et al. [28]. 0.75 mL of a methanol solution of each extract at different aliquot concentrations was mixed with 1.5 mL of DPPH methanol solution (0.5 mM). Mixtures were vigorously shaken and left 30 min in the dark at room temperature. The absorbance was then measured at 517 nm. BHT and gallic acid were used as positive references while methanol was used as negative one. Inhibition of DPPH radical was calculated as follows:

(1) $$\text{DPPH}\;\text{radical}\;\text{inhibition}\;\text{\%}=\left(1-\frac{A_{\text{test}}}{A_{\text{control}}}\right)\times 100,$$

where *A*_test_ was the absorbance of the test sample (DPPH solution plus antioxidant) and *A*_control_ was the absorbance of the control (DPPH solution without test sample). IC_50_ value, which represented the concentration of the extract that caused 50% inhibition, was calculated by linear regression module of Prism 5.0.

#### 2.2.3. Ferric Reducing Antioxidant Power (FRAP) Assay

Total antioxidant activity was investigated using Ferric Reducing Antioxidant Power (FRAP) assay according to the method of Oyaizu [29]. FRAP reagent was freshly prepared by mixing 1 mL sodium phosphate buffer (0.2M, pH 6.6) and 1.0 mL 1% potassium ferricyanide (K_3_Fe(CN)_6_). 150 *μ*L of each sample (1.0 mg/mL) dissolved in methanol was added in 4.5 mL of FRAP reagent, stirred, and incubated at 50°C for 20 min. Afterwards, 1.0 mL 10% trichloroacetic acid was needed, followed by centrifugating at 3000 rpm for 10 min. Finally, the supernatant solution (2.5 mL) was mixed with 2.5 mL of distilled water and 0.5 mL of 0.1% FeCl_3_, and the absorbance was measured at 700 nm. Higher absorbance of the reaction mixture indicated greater reducing power. Calibration curve of ferrous sulfate (100–1000 mmol/L) was used, and the results were expressed in *μ*mol Fe^2+^/mg dry weight extract. The relative activity of the samples was compared to butylated hydroxytoluene (BHT) and gallic acid. 

#### 2.2.4. Thiobarbituric Acid (TBA) Assay

The method of Ottolenghi was used to determine the TBA (thiobarbituric acid) values of the samples [30]. The formation of malonaldehyde, a secondary breakdown product of lipid hydroperoxides, is the basis for the well-known TBA method used for evaluating the extent of lipid peroxidation (LP), which is the process that involves the chain reaction of free radicals with polyunsaturated fatty acids. In this study, the extracts in concentrations of 100 *μ*g/mL were mixed with 300 *μ*L Tris-HCl buffer, pH 7.5, 500 *μ*L of 20 mM linoleic acid, and 100 *μ*L of 4 mM FeSO_4_. The peroxidation was started with addition of 100 *μ*L 5 mM ascorbic acid. The reaction mixture was incubated for 1 h at 37°C. Then, 2 mL of 20% trichloroacetic acid (CCl_3_COOH) and 2 mL TBA aqueous solution were added to 1 mL of sample solution prepared in different concentrations, incubated in a similar manner. The mixture was then heated in a boiling water bath for 10 min. After cooling on ice, it was centrifuged at 3000 rpm for 20 min and the absorbance of the supernatant was measured at 532 nm. Inhibition of LP was calculated the same way as described in DPPH radical assay. And the same controls mentioned above were used.

### 2.3. Determination of Cytotoxic Activity

#### 2.3.1. Cell Culture and Treatments

SMMC-7721 cells were obtained from Cancer Cell Repository (The First Affiliated Hospital, Zhejiang University. China). The cells were grown and maintained in a humidified incubator at 37°C under 5% CO_2_ atmosphere in RPMI 1640 medium, supplemented with 10% fetal calf serum, 100 U/mL penicillin, and 100 *μ*g/mL streptomycin. For experiments, cells were plated in 96-well plates (at a density of 5 × 10^4^ cells/mL). After 24 h incubation to allow cell attachment, the cells were treated with fresh medium containing different concentrations of extracts of *A. macrosperma* dissolved in DMSO (1%) and incubated for 48 h under the same conditions. Control groups received the same amount of DMSO. Quercetin was used as a positive control.

#### 2.3.2. MTT Bioassay

The MTT colorimetric assay, which is based on the reduction of MTT [3-(4,5-dimethyl-2-thiazolyl)-2,5-diphenyl-*2H*-tetrazolium bromide] by mitochondrial dehydrogenase to a purple formazan product, was used to assess the antiproliferative action of* A. macrosperma* extracts in human hepatoma SMMC-7721 cells. At the end of 48 h incubation, the medium in each well was added by 10 *μ*L MTT solution (0.5 mg/mL in PBS) and incubated for another 4 h. The supernatant was then removed and replaced with 200 *μ*L of DMSO to dissolve the resulting MTT formazan crystals, followed by mixing and measuring the absorbance using a microplate spectrophotometer (Bio-Rad, Model 550, USA) at 590 nm. The effect was quantified as the percentage of control absorbance of reduced dye as follows:

(2) $$\text{Inhibition\%}=\left[1-\frac{\left(A_{\text{test}}-A_{\text{blank}}\right)}{\left(A_{\text{control}}-A_{\text{blank}}\right)}\right]\times 100,$$

where *A*_test_, *A*_blank_, and *A*_control_ were the absorbance of the test sample, blank, and control, respectively. Before treating a specific cell line, the range of linearity for the MTT assay (extinction versus cell number) was defined. In the experiment, 6 wells were used for each concentration. Each experiment was repeated two to three times. IC_50_ (50% inhibitory concentration) were determined by nonlinear regression analysis using Prism 5.0 software.

### 2.4. Statistical Analysis

All the analyses were carried out in triplicate, and the results were expressed as the mean ± SEM. Statistical analyses were performed by *t*-test or ANOVA, The difference was considered significant if *P* < 0.05.

## 3. Results

### 3.1. Amount of Total Phenolic Content

It has been reported that the antioxidant activity of plant materials was well correlated with the content of their phenolic compounds [31]. The total phenolic amount in the hexane, ethyl acetate, methanol, aqueous, and chloroform extracts of *A. macrosperma* stems was shown in Table 1. All extracts contained a considerable amount of phenolic metabolites from 5.11 mg/g to 114.10 mg/g (gallic acid equivalent). The highest was the MeOH extract, followed by the EtOAc, aqueous, CHCl_3_, and hexane extracts in the decreasing order. The Folin-Ciocalteu procedure is nonspecific because it detects all phenolics (phenolic acids, flavonoids, and tannins) [32], so it does not give details of the quantity and quality of the phenolic constituents of the extracts. Nevertheless, this widely used method provides a rapid and useful overall evaluation of the phenolic content of extracts. 

### 3.2. DPPH Radical Scavenging Activity

The free radical scavenging activity of *A. macrosperma* growing in China was determined using a stable DPPH free radical. The DPPH test intends to measure the hydrogen atom or electron donor capacity of the extracts to the stable radical DPPH formed in solution [33]. The DPPH reagent evidently offered a convenient and accurate method for measuring the capacity of the extract to scavenge free radicals in solution. The DPPH radical scavenging activities of the known antioxidative substances (BHT and gallic acid) and the extracts studied in this paper were shown in Table 2. 

In DPPH assay, dose-dependent inhibition was evaluated in all extracts. Concentrations at which extracts decrease DPPH radical by 50% (IC_50_ values) were 1091.00 *μ*g/mL, 52.90 *μ*g/mL, 40.66 *μ*g/mL, 264.00 *μ*g/mL, and 621.40 *μ*g/mL for hexane, EtOAc, MeOH, aqueous, and CHCl_3_ extracts, respectively. IC_50_ values for BHT and gallic acid, used as standards, were 44.07 and 4.62 *μ*g/mL. The result showed the EtOAc and MeOH extract of *A. macrosperma* possessed significant DPPH radical scavenging activity as evidenced by low IC_50_ values, similar to that of BHT, but lower than that of gallic acid. Moreover, no significant difference was observed among EtOAc and MeOH extract. By contrast, the inhibition of aqueous and CHCl_3_ extracts was more slight. On the other hand, more than 1000 *μ*g/mL of hexane extract was necessary to achieve the same results.

### 3.3. Reducing Power

FRAP values for investigated extracts are shown in Table 2. The reducing powers of all extracts, BHT and gallic acid increased with the concentration. The reducing power of MeOH extract was more pronounced than those of other extracts, and it was similar to that of BHT. The EtOAc, aqueous, and CHCl_3_ extracts showed similar appreciable activity (*P* > 0.05), but the hexane did slightly. All extracts had TAA lower than gallic acid used as standard (7.85 *μ*mol Fe^2+^/mg). The reducing power of a compound was related to its electron transfer ability and may, therefore, serve as an indicator of its potential antioxidant activity [34]. Moreover, extracts with phenolic substance-mediated antioxidant activity were shown to be concomitant with the development of reducing power [35], thus, the MeOH extract contained higher amounts of reducing compounds which were electron donors and could react with free radicals and convert them to more stable products and terminate radical chain reaction [36].

### 3.4. Inhibition of Lipid Peroxidation (LP)

In TBA method, malonaldehyde binds TBA to form a red complex that can be measured at 532 nm. The increase of the amount of red pigment formed correlates with the oxidative rancidity of the lipid. The *A. macrosperma* extracts had an inhibition of LP in a similar, concentration-dependent manner, which lowered the degree of LP induced by hydroxyl radical generated by an iron/ascorbate system. IC_50_ values were given in Table 2. Based on the results, the inhibition was highest in gallic acid and BHT with IC_50_ of 0.89 *μ*g/mL and 20.81 *μ*g/mL, respectively, followed by MeOH (24.83 *μ*g/mL), EtOAc (29.45 *μ*g/mL), CHCl_3_ (48.67 *μ*g/mL), and aqueous (61.32 *μ*g/mL) extract, while hexane extract did not reach 50% of LP inhibition even at the highest concentration. 

### 3.5. Cytotoxic Activity

In Table 3, the hexane, EtOAc, and CHCl_3_ extracts of *A. macrosperma* showed cytotoxicity against human hepatoma SMMC-7721 tumor cell lines (IC_50_ < 100 *μ*g/mL) with a dose-dependent manner. Methanol and aqueous extracts, however, showed weak activities (IC_50_ > 180 *μ*g/mL) in this test. Thereinto, a very significant cytotoxic activity, not significantly different from the positive control of quercetin (*P* > 0.05), was observed in CHCl_3_ extract (IC_50_ = 50.97 *μ*g/mL). Since the plant was extracted successively with hexane, EtOAc, and MeOH, a slight cytotoxic activity of the ethyl acetate extract indicated that the cytotoxic compound in the hexane extract differed from that in the methanol extract.

## 4. Discussion

Safety is an essential consideration for antioxidants as they may be utilized in the manufacture of foods and pharmaceuticals. Interest in the search for new natural antioxidants has grown dramatically over the past years. *Actinidia* species have been investigated particularly as a source of natural antioxidants [37, 38]. They are good sources of several vitamins and minerals and dietary fibre, and contain a number of phytochemicals. These beneficial phytochemicals often have antioxidant effects, assumed to be involved in scavenging excess free radicals that arise not only as a result of stress and disease but also as a part of normal metabolic processes [39]. The results obtained in this work are noteworthy, not only with respect to the antioxidant and anticancer activity of *A. macrosperma* extracts, but also with respect to the confirmation of high correlations between total phenolics content and the antioxidant activity.

In view of the differences among the test systems available, the results of a single assay can give only a suggestion on the protective potential of phytochemicals. Therefore, the use of more than one method is highly advisable. Among the methods that can be used for the evaluation of the antioxidant activity, few of them (TEAC, DPPH, PCL, ORAC) are useful to determine the activity of both hydrophilic and lipophilic species, thus ensuring a better comparison of the results [40]. This study indicated that different polarity extracts of *A. macrosperma* exhibit different biological activities, which depends mainly on the presence of phenolic compounds. The antioxidant activity was in the following decreasing order: Gallic acid > MeOH extract > BHT > EtOAc extract > aqueous extract > CHCl_3_ extract > Hexane extract. The methanol extract from the stem of* A. macrosperma* showed the most potent antioxidant activity based on DPPH, reducing power, and TBA tests in a dose-dependent manner, similar to that of BHT. It strongly scavenged DPPH radicals with the IC_50_ being 40.66 ± 2.42 *μ*g/mL, inhibited the LP with IC_50_ of 24.83 ± 0.22 *μ*g/mL, and also caused significant elevation of reducing power (FRAP value was 1.327 ± 0.059). 

Plants phenolic compounds constitute one of the major groups of compounds acting as primary antioxidants and free radical terminators by donating hydrogen from the phenolic hydroxyl groups [41]. In this experiment, MeOH and EtOAc extracts had the highest polyphenols content (114.10 and 70.46 mg gallic acid equivalent/g), as well as the highest antioxidant activities (Table 2). Hexane extract had the least polyphenols (5.11 mg gallic acid equivalent/g) and had minimum of antioxidant activities (Table 2). All extracts had antioxidant activities lower than gallic acid used as standard. But except in TBA test, no significant difference was found between the total antioxidant activity of MeOH extract compared with BHT (another standard) in both methods. Therefore, antioxidant activity which showed up in this assay was generally associated with more polar constituents such as phenolics. Content of total phenolics was correlated with the result of DPPH radical assay and the results obtained in the FRAP and TBA assay. The presence of phenolic compounds in the extracts might be responsible for the antioxidant activity found in the present study, due to their redox properties of absorbing and neutralizing free radicals, quenching singlet and triplet oxygens, or decomposing peroxides [15]. This result was in agreement with findings of prior studies carried out on other different herbal extracts [42, 43]. However, in the evaluation of cytotoxic activity, methoxyl substitutions were responsible for the cytotoxic activity in hexane, EtOAc, and CHCl_3_ extracts. This indicated that there was no relationship between antitumor and antioxidant activities. 

The high antioxidant potential of *A. macrosperma* can broaden their therapeutic applications towards the prevention of degenerative and neoplastic diseases of various organs. Human cells are constantly exposed to reactive oxygen radicals generated by a number of biotic and abiotic factors such as irradiation, atmospheric and food pollutants or byproducts of metabolic processes. When the exposure overwhelms endogenous preventive systems, cells are exposed to a potentially harmful load of oxidants, leading to various free-radical-induced noxious effects. These include, among others, oxidation of nucleic acids, proteins, lipids, and carbohydrates, which may subsequently determine mutagenesis and diseases related to DNA damage [44]. Thus, the medicinal claims of this plant being used in the treatment of liver disorders, tumours, and abscess may be in part due to the antioxidant activity. This hypothesis requires wider and detailed studies for its potential importance for the more directed search of new natural sources of powerful antioxidants for therapeutic use. Further activity-guided isolation and characterization of antioxidant or antitumor compounds and in vivo antioxidant or antitumor studies are still in progress.

## Figures and Tables

**Table 1:** Total phenolic content in the extracts of *A. macrosperma* (gallic acid equivalent).

Extract	Phenolics (mg gallic acid equivalents/g extract)
Hexane	5.11 ± 0.13^e^
EtOAc	70.46 ± 1.01^b^
MeOH	114.10 ± 0.71^a^
Aqueous	57.06 ± 2.26^c^
CHCl_3_	13.73 ± 0.76^d^

Means with the same letter are not sinificantly different at *P* < 0.05.

**Table 2:** Antioxidant activities of the different extracts of *A. macrosperma. *

Extract/control	DPPH radical scavenging activity IC_50_ (*μ*g/mL)	FRAP value^1^	Inhibitory effect of LPIC_50_ (*μ*g/mL)
Hexane	1091.00 ± 75.61^a^	0.224 ± 0.024^d^	—
EtOAc	52.90 ± 1.65^d^	0.872 ± 0.025^c^	29.45 ± 0.29^c^
MeOH	40.66 ± 2.42^d^	1.327 ± 0.059^b^	24.83 ± 0.22^d^
Aqueous	264.00 ± 16.49^c^	0.617 ± 0.026^c^	61.32 ± 0.46^b^
CHCl_3_	621.40 ± 16.49^b^	0.462 ± .0228^c^	48.67 ± 0.53^a^
BHT	44.07 ± 4.58^d^	1.384 ± 0.055^b^	20.81 ± 0.52^e^
Gallic acid	4.62 ± 0.33^e^	7.850 ± 0.215^a^	0.89 ± 0.04^f^

Means in one column with the same letter are not significantly different at *P* < 0.05.

^1^In units *μ*mol Fe^2+^/mg dry weight extract.

— means no activity.

**Table 3:** Antiproliferative activity of *A. macrosperma* extracts in SMMC-7721 cells.

Extract	IC_50_ (*μ*g/mL)
Hexane	70.60 ± 2.17^c^
EtOAc	192.00 ± 2.41^b^
MeOH	76.61 ± 3.37^c^
Aqueous	212.10 ± 4.63^a^
CHCl_3_	50.97 ± 1.28^d^
Quercetin	44.32 ± 1.61^d^

Means with the same letter are not significantly different at *P* < 0.05.

## References

[1] Halliwell B (1994). Free radicals, antioxidants and human disease: curiosity, cause or consequence. *The Lancet*.

[2] Finkel T, Holbrook NJ (2000). Oxidants, oxidative stress and the biology of ageing. *Nature*.

[3] Sang S, Lapsley K, Jeong WS, Lachance PA, Ho CT, Rosen RT (2002). Antioxidative phenolic compounds isolated from almond skins (*Prunus amygdalus* Batsch). *Journal of Agricultural and Food Chemistry*.

[4] Touyz RM (2003). Reactive oxygen species in vascular biology: role in arterial hypertension. *Expert Review of Cardiovascular Therapy*.

[5] Emmons CL, Peterson DM, Paul GL (1999). Antioxidant capacity of oat (*Avena sativa* L.) extracts. 2. In vitro antioxidant activity and contents of phenolic and tocol antioxidants. *Journal of Agricultural and Food Chemistry*.

[6] Rice-Evans CA, Sampson J, Bramley PM, Holloway DE (1997). Why do we expect carotenoids to be antioxidants in vivo?. *Free Radical Research*.

[7] Lu Y, Yeap Foo L (2000). Antioxidant and radical scavenging activities of polyphenols from apple pomace. *Food Chemistry*.

[8] Ito N, Hirose M, Fukushima S, Tsuda H, Shirai T, Tatematsu M (1986). Studies on antioxidants: their carcinogenic and modifying effects on chemical carcinogenesis. *Food and Chemical Toxicology*.

[9] Lean LP, Mohamed S (1999). Antioxidative and antimycotic effects of turmeric, lemon-grass, betel leaves, clove, black papper leaves and Garcinia atriviridis on butter cackes. *Journal of the Science of Food and Agriculture*.

[10] Zia-ur-Rehman, Salariya AM, Habib F (2003). Antioxidant activity of ginger extract in sunflower oil. *Journal of the Science of Food and Agriculture*.

[11] Brevik A, Gaivão I, Medin T (2011). Supplementation of a western diet with golden kiwifruits (*Actinidia chinensis* var. ’Hort 16A’:) effects on biomarkers of oxidation damage and antioxidant protection. *Nutrition Journal*.

[12] Shahidi F, Janitha PK, Wanasundara PD (1992). Phenolic antioxidants. *Critical Reviews in Food Science and Nutrition*.

[13] Velioglu YS, Mazza G, Gao L, Oomah BD (1998). Antioxidant activity and total phenolics in selected fruits, vegetables and grain products. *Journal of Agricultural and Food Chemistry*.

[14] Pietta P, Simonetti P, Mauri P (1998). Antioxidant activity of selected medicinal plants. *Journal of Agricultural and Food Chemistry*.

[15] Osawa T, Uritany I, Garcia VV, Mendoza EM (1994). Novel natural antioxidants for utilization in food and biological systems,. *Postharvest Biochemistry of Plant Food-Materials in the Tropics*.

[16] Jiangsu New Medicine College (1984). *Dictionary of Chinese Traditional Medicines*.

[17] Liang CF, Chen YC, Wang YS (1984). *Flora of China*.

[18] Lai PF, Zhang HY (2002). The research progress of TCM Cat Ginseng in Zhejiang province location. *Journal of Zhejiang College of TCM*.

[19] Yao G, Wang TS (1989). Medicinal plants of *Actinidia* genus in East China. *Journal of Chinese Medicinal Materials*.

[20] Zhou XZ (2000). Tang Fu’an talk about lung cancer treatment variation. *Journal of Zhejiang College of TCM*.

[21] Wan XY, Zhang YN, Zhang C (2004). Experimental study on Antihepatocarcinoma effect of Maorenshen injection in vitro. *Journal of Zhejiang College of TCM*.

[22] Zhang YN, Liu L, Ling CQ (2006). Inhibition effect of active fraction from Maorenshen on growth of transplanated mouse tumor cells and preliminary study of its mechanism. *Journal of Chinese Medicinal Materials*.

[23] Jiang WM, Li FY (2003). Establishement of plantlet regeneration system of *Actinidia macrosperma*. *Journal of Zhejiang University*.

[24] Lu Y, Fan J, Zhao Y (2007). Immunomodulatory activity of aqueous extract of *Actinidia macrosperma*. *Asia Pacific Journal of Clinical Nutrition*.

[25] Lu Y, Zhao YP, Wang ZC, Chen SY, Fu CX (2007). Composition and antimicrobial activity of the essential oil of *Actinidia macrosperma* from China. *Natural Product Research*.

[26] Zhao YP, Zhong YJ, Lu Y, Wang ZC, Chen SY, Fu CX (2006). Pharmacognostic identification on stems of nine *Actinidia* species. *Chinese Pharmaceutical Journal*.

[27] Lu Y, Zhao YP, Fu CX (2011). Biological activities of extracts from a naturally wild kiwifruit, *Actinidia macrosperma*. *African Journal of Agricultural Research*.

[28] Brand-Williams W, Cuvelier ME, Berset C (1995). Use of a free radical method to evaluate antioxidant activity. *Lebensm Wiss Techonol*.

[29] Oyaizu M (1986). Studies on product of browning reaction prepared from glucose amine. *Japanese Journal of Nutrition*.

[30] Ottolenghi A (1959). Interaction of ascorbic acid and mitochondria lipids. *Archives of Biochemistry and Biophysics*.

[31] Velioglu YS, Mazza G, Gao L, Oomah BD (1998). Antioxidant activity and total phenolics in selected fruits, vegetables, and grain products. *Journal of Agricultural and Food Chemistry*.

[32] Niklová I, Schmidt Š, Habalová K, Sekretár S (2001). Effect of evening primrose extracts on oxidative stability of sunflower and rapeseed oils. *European Journal of Lipid Science and Technology*.

[33] Tepe B, Daferera D, Sokmen A, Sokmen M, Polissiou M (2005). Antimicrobial and antioxidant activities of the essential oil and various extracts of *Salvia tomentosa* Miller (Lamiaceae). *Food Chemistry*.

[34] Sanchez-Moreno C (2002). Methods used to evaluate the free radical scavenging activity in foods and biological systems. *Food Science and Technology International*.

[35] Siddhuraju P, Becker K (2003). Studies on antioxidant activities of mucuna seed (*Mucuna pruriens var utilis*) extract and various non-protein amino/imino acids through in vitro models. *Journal of the Science of Food and Agriculture*.

[36] Chung YC, Chang CT, Chao WW, Lin CF, Chou ST (2002). Antioxidative activity and safety of the 50% ethanolic extract from red bean fermented by *Bacillus subtilis* IMR-NK1. *Journal of Agricultural and Food Chemistry*.

[37] Latocha P, Krupa T, Wołosiak R, Worobiej E, Wilczak J (2010). Antioxidant activity and chemical difference in fruit of different *Actinidia* sp.. *International Journal of Food Sciences and Nutrition*.

[38] Xin HL, Wu YC, Su YH, Sheng JY, Ling CQ (2011). Novel flavonoids from the leaves of *Actinidia valvata* Dunn: structural elucidation and antioxidant activity. *Planta Medica*.

[39] Skinner MA, Loh JMS, Hunter DC, Zhang JL (2011). Antioxidants and the immune system Gold kiwifruit (*Actinidia chinensis* ‘Hort16A’) for immune support. *Proceedings of the Nutrition Society*.

[40] Prior RL, Wu X, Schaich K (2005). Standardized methods for the determination of antioxidant capacity and phenolics in foods and dietary supplements. *Journal of Agricultural and Food Chemistry*.

[41] Dziezak JD (1986). Antioxidants. *International Journal of Food Science & Technology*.

[42] Miliauskas G, Venskutonis PR, Van Beek TA (2004). Screening of radical scavenging activity of some medicinal and aromatic plant extracts. *Food Chemistry*.

[43] Yildirim A, Mavi A, Kara AA (2003). Antioxidant and antimicrobial activities of *Polygonum cognatum* Meissn extracts. *Journal of the Science of Food and Agriculture*.

[44] Ames BN, Shigenaga MK, Hagen TM (1993). Oxidants, antioxidants, and the degenerative diseases of aging. *Proceedings of the National Academy of Sciences of the United States of America*.

